# Correlation between temporomandibular joints and craniocervical posture in patients with bilateral anterial disc displacement

**DOI:** 10.1186/s12903-024-03892-9

**Published:** 2024-02-01

**Authors:** Wanfang Xiang, Min Wang, Zhihui Li, Mingqin Cai, Xiaojing Pan

**Affiliations:** 1 School/Hospital of Stomatology, Lanzhou University, Tianshui South Road, Chengguan District, Lanzhou, Gansu Province 730000 People’s Republic of China; 2 School of Basic Medical Sciences, Lanzhou University, Lanzhou, 730000 People’s Republic of China

**Keywords:** Temporomandibular joint (TMJ), Craniocervical posture, Anterior disc displacement (ADD), Morphometric measurements

## Abstract

**Objective:**

To study the changes of temporomandibular joints and craniocervical posture in adult patients with bilateral anterior disc displacement, and to explore their correlation, which may provide some clinical value for clinical diagnosis and treatment planning.

**Methods:**

Ninety-eight adult patients were divided into 3 groups: 29 patients in bilateral disc normal position group (BN), 33 patients in bilateral Anterior Disc Displacement With Reduction group (ADDWR) and 36 patients in bilateral Anterior Disc Displacement Without Reduction group (ADDWoR). Dolphin and Uceph software were used to measure 14 items of temporomandibular joint and 11 items of craniocervical posture for comparison and correlation analysis between groups.

**Results:**

There were significant differences in bilateral joint space between three groups. Compared with the BN, the anteroposterior diameter of the condyle was significantly reduced, the condyle was significantly displaced posteriorly and superiorly in the ADDWR and ADDWoR, but the joint fossa width and joint fossa depth did not change significantly. Cervical curvature and inclination were greater in patients with anterior disc displacement than BN, indicating that the craniocervical posture of adult patients with anterior disc displacement was extended and protrusive.

**Conclusion:**

Anterior disc displacement of the temporomandibular joint can displace the condyle upwards and posteriorly and reduce the anteroposterior diameter of condyle, and then make the condyle closer to the wall of articular fossa to induce joint symptoms. Additionally, craniocervical postural position is significantly affected, which may be related to compensate for the effects of airway space.

**Supplementary Information:**

The online version contains supplementary material available at 10.1186/s12903-024-03892-9.

## Highlights

1.In individuals with bilateral anterior disc displacement, the majority of temporomandibular joint abnormalities are asymmetrical, whereas in those with normal disc position, the bilateral temporomandibular joint is frequently symmetrical.

2.It was firstly determined how the bilateral TMJs in patients with different stages of ADD changed in three dimensions dynamically.

3.Patients with bilateral anterior disc displacement often have cervical vertebra anteversion and enlarged cervical curvature and Anterior disc displacement may lead to increased craniocervical posture somehow, and vice versa.

**Supplementary Information:**

The online version contains supplementary material available at 10.1186/s12903-024-03892-9.

Temporomandibular disorders (TMDs) are a group of musculoskeletal diseases that involve the temporomandibular joints (TMJs), the masticatory muscles and all associated tissues [1]. TMDs are a group of common complex oral diseases, which present with pain and functional limitation of the temporomandibular joint (TMJ) [2–4]. Numerous studies have shown that anterior disc displacement (ADD) is the most common structural disorder leading to TMD symptoms, and can be divided into Anterior Disc Displacement With Reduction (ADDWR) and Anterior Disc Displacement Without Reduction (ADDWoR) [5]. In recent years, the potential correlation between ADD and craniocervical posture has gradually become a frontier research issue [6–9].

At present, most studies are limited to a single anterior disc displacement stage, and relevant literature investigating the dynamic development of anterior disc displacement is rare. Therefore, the aim of this study was to quantitatively investigate the three-dimensional dynamic changes of bilateral TMJ morphology and position at different stages of bilateral anterior disc displacement by CBCT, and to analyze their craniocervical posture differences with normal craniocervical posture, and further to investigate the correlation between temporomandibular joints and craniocervical posture.

## Materials and methods

### Study subjects

**Fig. 1:**
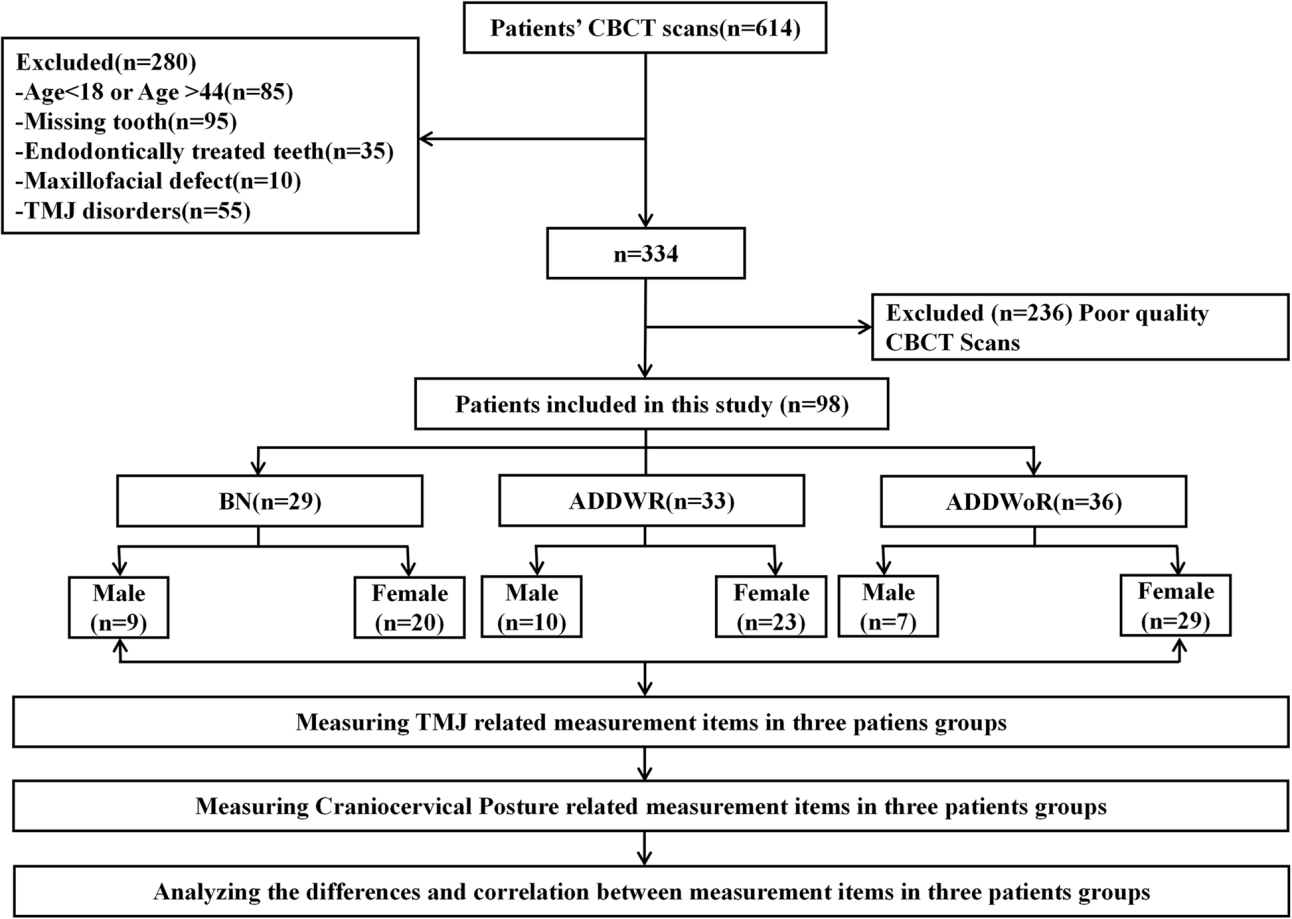
Experimental procedure flowchart (Inclusion and exclusion criteria refer to Supplementary table S1)

A cross-sectional retrospective study was designed and implemented to reasonably address the research purpose. Patients sufered with ADDWR or ADDWoR admitted to the Temporomandibular Joint Specialist Clinic, Lanzhou University Stomatology Hospital, China, from January 2020 to December 2022 constituted the study population (Fig. 1). The approved protocol (LZUKQ-2023–041) was subscribed by Ethics Committee for Clinical Scientific Research of Lanzhou University School of Stomatology.

### Investigational equipment

All subjects underwent CBCT scan and lateral cephalogram in the Department of Radiology, Stomatology Hospital of Lanzhou University. The products models were ORTHOPHOSSL3D (Sirona Dental System GmbH, Germany) and KaVo OP 3D Vision (Imaging Sciences international, United States). During the process of taking images, all subjects were required to sit straight, keep Frankfort horizontal plane parallel to the ground, keep intercuspal position (ICP), don’t swallow and hold the breath till the process was done. Making sure that all participants were photographed under the posture of natural head position.

### Measuring items

Based on the software Dolphin Imaging 11.8 (Chatsworth, California) and Uceph 4.2.1 (Chengdu, Sichuan), the measurement items related to TMJ and craniocervical posture were measured.

#### Three-dimensional measurement items of the temporomandibular joint (Figs. 2, 3, 4)

**Fig. 2:**
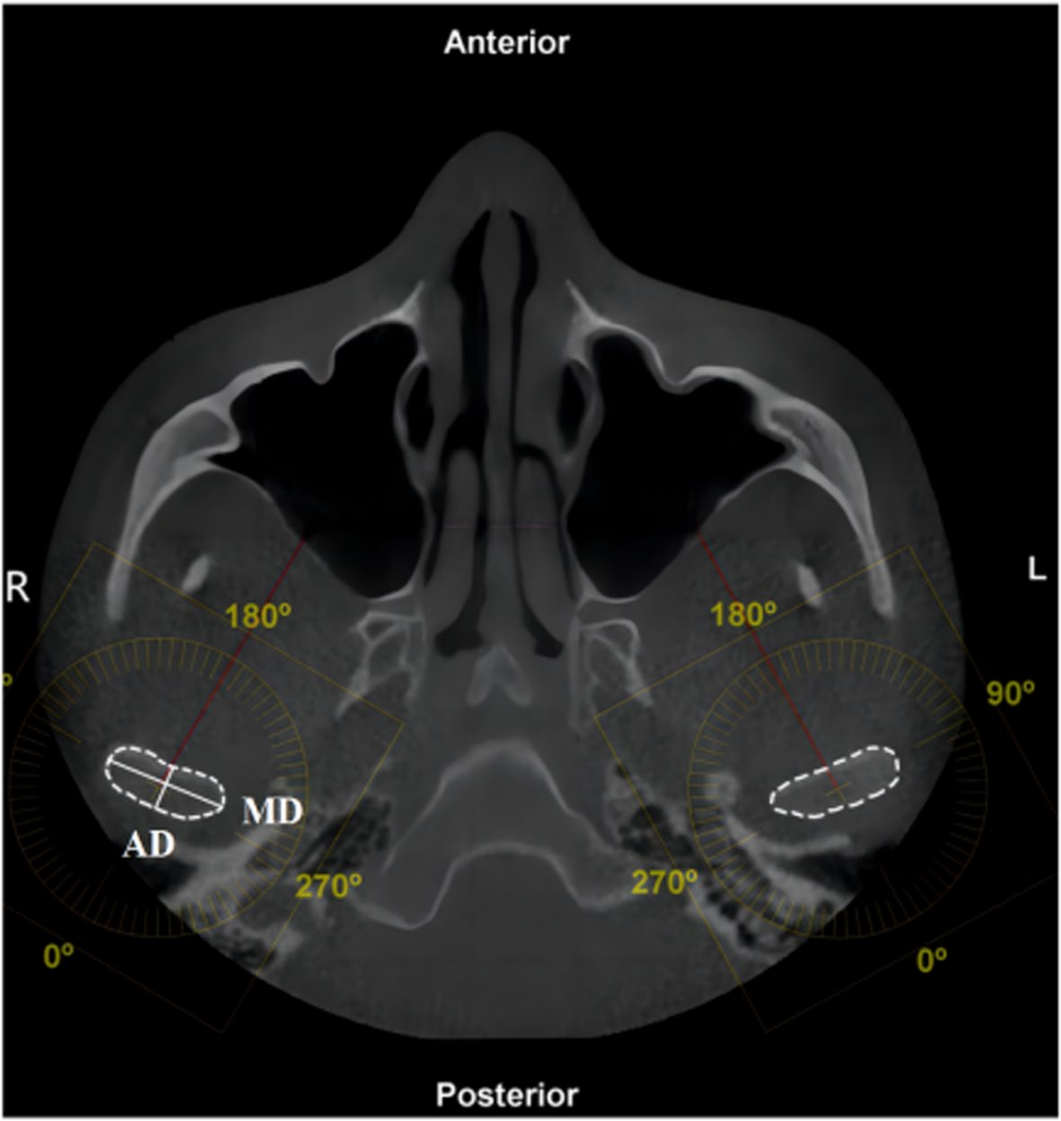
Axial measurement items 1. Condyle inner and outer diameter (MD, the distance between the innermost point and the outermost point of the condyle); 2. Anteroposterior diameter of condyle (AD, the distance between the anterior point and the posterior point of condyle)

**Fig. 3:**
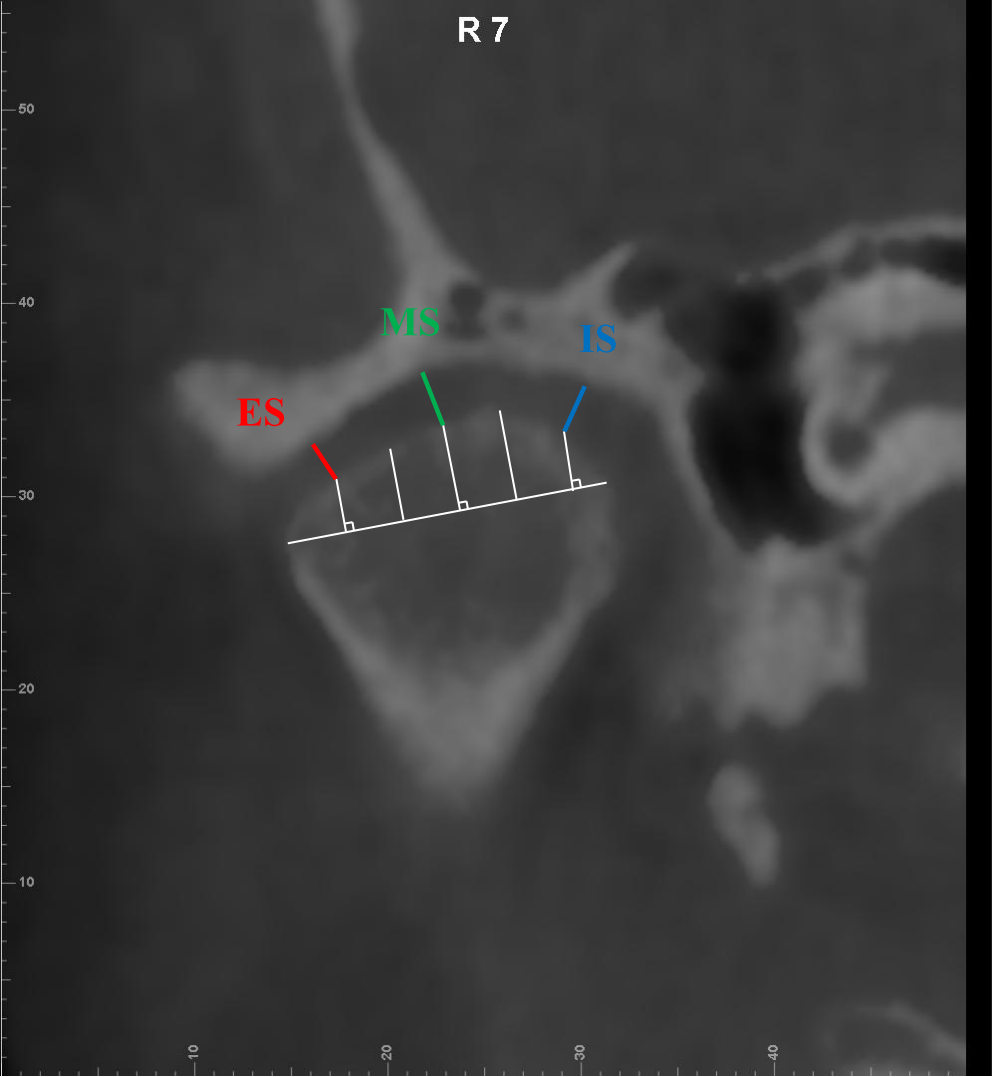
Coronal measurement items 1.Intra-articular space (IS); 2. Middle joint space (MS); 3. Extra-articular space (ES). Note: The width of the condyle was divided into six equal parts. The shortest distance from the point where the vertical line intersected with the condyle to the articular fossa at the first medial part is IS. The shortest distance from the point where the vertical line intersected with the condyle to the articular fossa at the midpoint part is MS. The shortest distance from the point where the vertical line intersected with the condyle to the articular fossa at the last medial part is ES

**Fig. 4:**
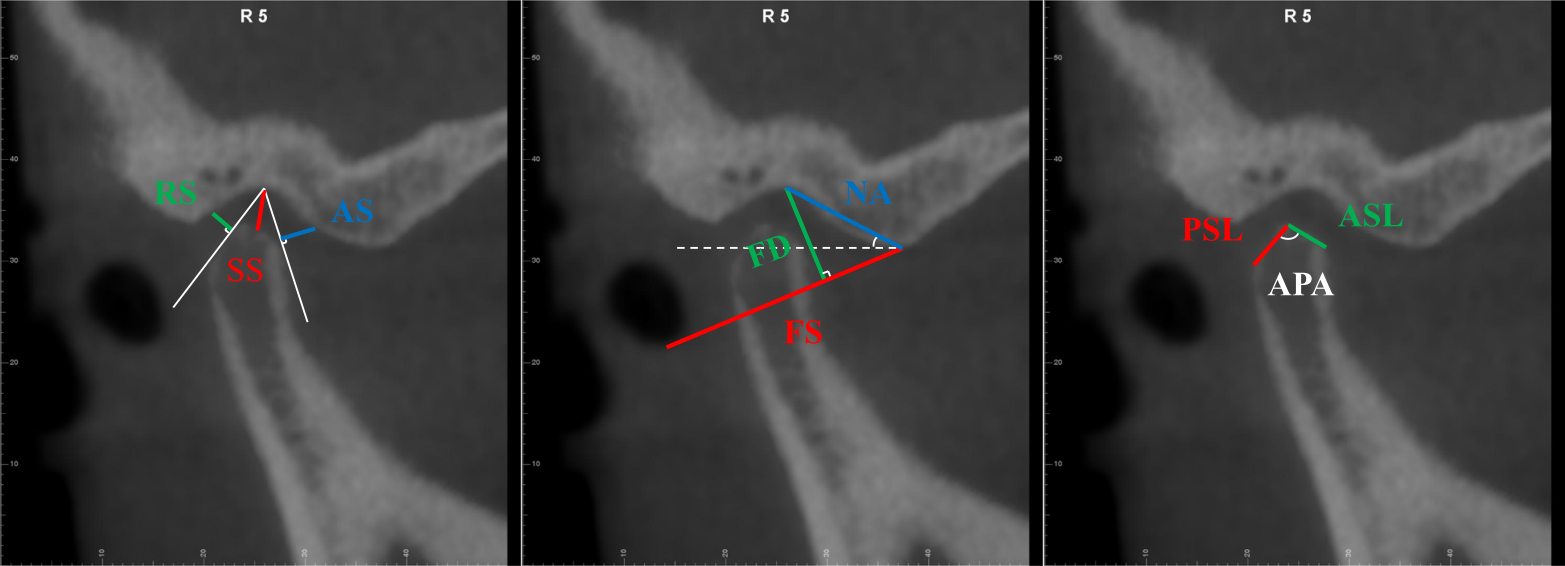
Sagittal measurement items 1. Anterior joint space (AS, the shortest distance between the anterior edge of the articular fossa and the tangent point from the apex of the articular fossa to the anterior edge of the condyle); 2. Superior joint space (SS, the shortest distance from the apex of the articular fossa to the apex of the condyle); 3. Posterior joint space (RS, the shortest distance between the posterior edge of the articular fossa and the tangent point from the apex of the articular fossa to the posterior edge of the condyle); 4. Joint fossa width (FS, the distance between the lowest points of the external auditory and articular tubercle); 5. Joint fossa depth (FD, the shortest distance between the apex of the articular fossa and the line of the articular fossa); 6. Joint tubercle angle (NA, the angle between the line connecting the apex of the articular fossa and the lowest point of the articular tubercle and Frankfort horizontal plane); 7. Length of anterior slope of condyle (ASL, the distance between the apex and the anterior point of condyle); 8. Length of posterior slope of condyle (PSL, the distance between the apex and the posterior point of condyle); 9. Angle between posterior and anterior slope of condyle (APA, the angle between the anterior slope and the posterior slope of condyle)

#### Craniocervical posture measurement items (Figs. 5, 6 and Table 1)

**Fig. 5:**
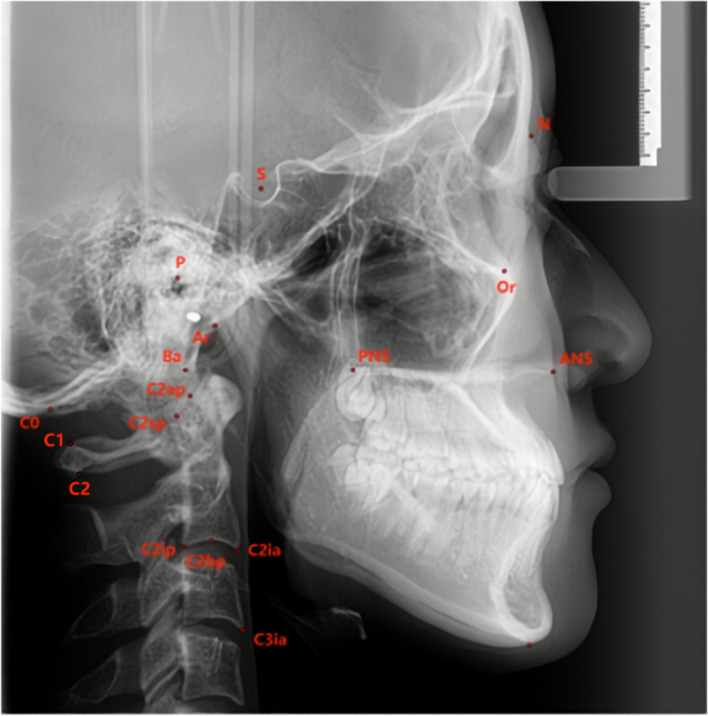
Landmarks used in this study: 1.N (naison); 2. S (sella); 3. Or (orbitale); 4. P (porion); 5. Ba (basion); 6. ANS (anterior nasal spine); 7. PNS (posterior nasal spine); 8. Ar (articulare); 9. C0 (the lowest point of the occipital squama); 10. C1 (the posterior arch of the Atlas); 11. C2 (the spinous process of the second cervical vertebra); 12. cv2sp (tangent point of the superoposterior extremity of the second cervical vertebra); 13. cv2ip (the most posteroinferior point on the second cervical vertebra); 14. cv2ia (the most anteroinferior point on the second cervical vertebra); 15. cv2ap (the apex of the second cervical vertebra); 16. cv2bp (middle point of lower edge of the second cervical vertebra); 17. cv3ia (the most anteroinferior point on the third cervical vertebra)

**Fig. 6:**
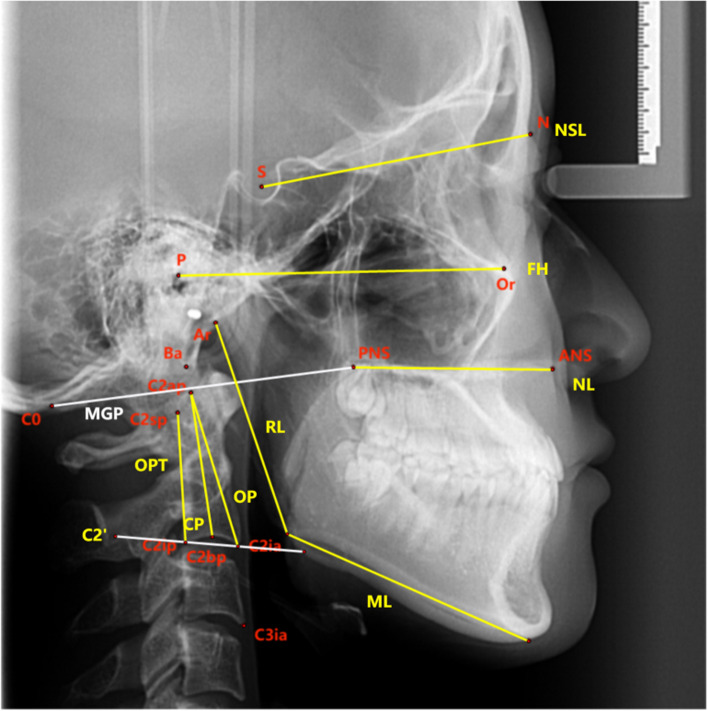
Craniocervical reference planes used in this study 1.Nasion-sella line (NSL, plane through nasion and sella); 2. Frankfort horizontal plane (FH, plane through porion and orbitale); 3. Nasal line (NL, line through the posterior nasal spine and anterior nasal spine); 4.Mandibular line (ML, a line tangent to the lower edge of the mandible); 5. Ramal line (RL, tangent of posterior margin of mandibular ramus); 6. Odontoid process tangent (OPT, the tangent of posterior second cervical vertebral); 7. OP (Odontoid plane, the tangent of anterior second cervical vertebral); 8. CP (Cervical plane, vertically bisect the line of the second cervical vertebral body); 9. C2’ (The tangent of the lower edge of the second cervical vertebra); 10. MGP (McGregor’s line, the line from the posterior edge of the hard palate to the lowest point of the occipital squama)

**Table 1:** Craniocervical posture measurement items and their definitions

	Measuring items	Definition
Linear Measurement	Ba-C3ia	The distance from the lowest anterior point of the third cervical vertebra (C3ia) to the midpoint of the anterior edge of the foramen magnum (Ba)
	C0-C1	The distance from the base of occipital bone (C0) to posterior arch of atlas (C1)
	C1-C2	The distance from the posterior arch of the atlas (C1) to the spinous process of the second vertebra (C2)
Angular Measurement	NSL-CP	The angle between cervical vertebra plane (CP) and anterior skull base plane (NSL)
	NSL-OPT	The angle between the odontoid tangent (OPT) and the anterior skull base plane(NSL)
	NL-OPT	The angle between the odontoid tangent (OPT) and the palatal plane (NL)
	FH-OPT	The angle between the odontoid tangent (OPT) and Frankfort horizontal plane (FH)
	ML-OPT	The angle between OPT and mandibular plane (ML)
	RL-OPT	The angle between the odontoid tangent (RL) and the mandibular ramus (RL)
	NSL-C2	The anterior superior angle between the anterior skull base plane (NSL) and the tangent line (C2) of the lower edge of the second cervical vertebra
	MGP-OP	The posterior-inferior angle of the intersection of the McGregor plane (MGP) and the odontoid plane (OP)

### Statistical analysis

PASS 15.0.5 was used to calculate the sample size power for this study. Statistical analysis was performed using the Statistical Product and Service Solutions, SPSS 22.0 (IBM, America). Shapiro–Wilk was used for normality test, Levene was used for homogeneity of variance test, LSD-t tests were used for each measurement to evaluate the average of differences between the sides for each element of the sample. All variables distributions were tested using Kruskal–Wallis when it didn’t obey normal distribution. When it obeyed normal distribution, ANOVA was used when variances were homogeneous; and Brown-Forsythe Anova was used when variances were heterogeneous. When the measurement data obeyed normal distribution, x ± S description and Pearson correlation analysis is employed, and when they didn’t obey normal distribution, M (P25, P75) description and Spearman correlation analysis is utilized.

## Results

The BN, ADDWR and ADDWoR groups patients were diagnosed by two joint specialists at joint specialty department of Stomatology Hospital of Lanzhou University during 2020y to 2022y. There were no joint symptoms in clinical examination or joint abnormalities in CBCT of BN group. Researchers themselves measured all items twice at an interval of one week under the same conditions to test the reliability. The sample size power for this study refer to Supplementary table S2.

### Patients’ general characteristics

A total of 98 subjects were included in this study (Table 2). Their age range was 18 to 44 (mean age, 26.5 ± 6.7 years). There were no significant differences in age and sex distribution among groups.

**Table 2:** Number and age distribution of subjects of BN, ADDWR and ADDWoR groups

Group	BN	ADDWR	ADDWoR	Total	Significance
Subjects,n(%)	29(30.1%)	33(33.6%)	36(36.7%)	98(100%)	NS
Sex(M,F)	9,20	10,23	7,29	26,72	NS
Age(y)					
Mean	25.3 ± 6.0	25.5 ± 6.5	28.9 ± 7.5	26.5 ± 6.7	NS
Range	19–36	18–41	19–44	18–44	NS

*NS* Not significant

### Symmetry of bilateral TMJ in BN group

There was no significant difference of the bilateral TMJ measurement items (*P* > 0.05), which indicted that the bilateral TMJ were basically symmetrical in morphology and location in healthy people (Table 3).

**Table 3:** Symmetry test of bilateral TMJ measurement items in BN group

Variable	(BN)		t or Z	Significance
	Left TMJ	Right TMJ		
MD	19.02 ± 2.61	18.80 ± 2.69	-0.729	NS
AD	7.31 ± 1.33	7.49 ± 1.35	-0.885	NS
IS	2.86 ± 0.61	2.99 ± 0.68	-0.950	NS
MS	2.70 ± 0.80	2.70 ± 0.71	1.778	NS
ES	2.27 ± 0.62	2.21 ± 0.59	0.456	NS
AS	2.01 ± 0.75	2.0 ± 0.60	0.397	NS
SS	3.51 ± 0.78	3.46 ± 0.90	0.467	NS
RS	2.20 ± 0.78	2.0 ± 0.72	0.888	NS
FS	27.19 ± 1.91	28.04 ± 2.34	-2.132	NS
FD	9.70 ± 4.27	9.70 ± 1.52	1.440	NS
NA	16.37 ± 7.78	16.94 ± 7.31	-0.736	NS
ASL	4.56 ± 0.95	4.80 ± 1.16	-1.147	NS
PSL	6.47 ± 1.58	7.27 ± 1.59	-3.131	NS
APA	94.76 ± 8.69	92.97 ± 10.03	1.201	NS

*NS* Not significant

### Symmetry of bilateral TMJ in ADDWR and ADDWoR group

**Fig. 7:**
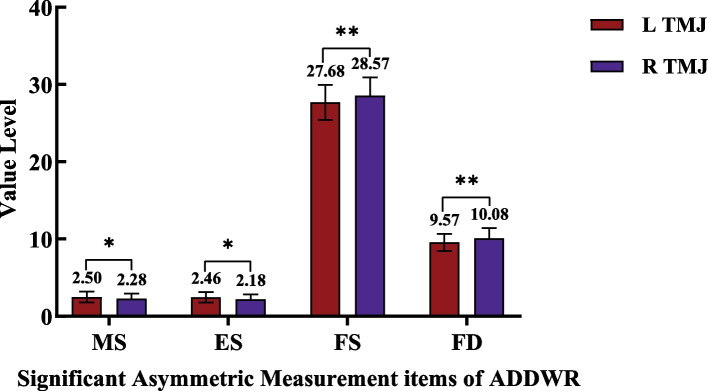
Bilateral TMJ significant asymmetric measurement items of ADDWR group 1.*P < 0.05; **P < 0.01; Multiple comparisons were used to analyze the intergroup difference at the level of α = 0.05

**Fig. 8:**
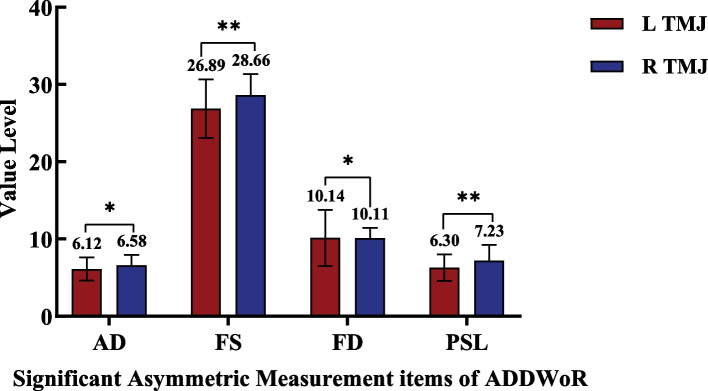
Bilateral TMJ significant asymmetric measurement items of ADDWoR group 1.*P < 0.05; **P < 0.01; Multiple comparisons were used to analyze the intergroup difference at the level of α = 0.05

In the ADDWR group, bilateral middle joint space (MS), extra-articular space (ES), fossa depth (FD), and fossa width (FS) were significantly different (*P* < 0.05). In the ADDWoR group, the bilateral condylar anteroposterior diameter (AD), the length of posterior slope of condyle (PSL), the joint fossa depth (FD) and joint fossa width (FS) were significantly different (*P* < 0.05). The results showed that in the process from ADDWR to ADDWoR, at the beginning the joint space asymmetry occurred and further developed into condylar morphology asymmetry, which may be due to the compensatory and protective effects of condylar cartilage. With the further development of ADD, condylar cartilage compensation became insufficient and organic changes appeared. Immediately after bilateral condylar destruction was inconsistent and there were differences between the left and right TMJ sides (Figs. 7 and 8).

### Bilateral TMJ changes in BN, ADDWR and ADDWoR groups

One-way anova analysis of bilateral TMJ measurement items in the BN, ADDWR and ADDWoR groups revealed that there were significant differences in the anteroposterior diameter (AD), anterior joint space (AS) and posterior joint space (RS) of the left and right TMJ (*P* < 0.05); There were significant differences in the intra-articular space (IS) and middle joint space (MS) of the right TMJ (*P* < 0.05); while there were no significant differences in the joint fossa depth (FD), joint fossa width (FS), joint nodular angle (NA), length of anterior slope or posterior of condyle (ASL, PSL) and angle between posterior and anterior slope of condyle (APA) of bilateral TMJ (*P* > 0.05). The results showed that during ADDWR developed into ADDWoR, the condylar morphology and joint space pathologically changed, but not articular fossa (Table 4).

**Table 4:** Bilateral TMJ changes in BN, ADDWR and ADDWoR groups

Variable	The Left TMJ				The Right TMJ			
	BN	ADDWR	ADDWoR	*P*	BN	ADDWR	ADDWoR	*P*
MD	18.900(17.2,20.2)	19.500(17.7,20.6)	18.050(16.9,19.6)	NS	18.91 ± 2.63	18.59 ± 2.71	17.83 ± 2.52	NS
AD	7.398 ± 1.332	6.988 ± 1.345	6.117 ± 1.494	0.000***	7.40 ± 1.33	6.88 ± 1.56	6.58 ± 1.37	0.020*
IS	2.92 ± 0.64	2.58 ± 1.11	2.68 ± 1.05	NS	2.92 ± 0.64	2.60 ± 0.94	2.47 ± 0.98	0.044*
MS	2.700(2.3,3.2)	2.500(1.9,3.0)	2.600(1.9,3.2)	NS	2.700(2.3,3.2)	2.300(1.9,2.8)	2.300(1.9,2.8)	0.003***
ES	2.24 ± 0.60	2.46 ± 0.67	2.40 ± 0.88	NS	2.250(1.7,2.6)	2.250(1.7,2.6)	2.250(1.7,2.6)	NS
AS	2.000(1.6,2.5)	3.800(2.6,4.7)	3.200(2.5,4.3)	0.000***	2.000(1.6,2.5)	2.000(1.6,2.5)	3.300(2.0,4.2)	0.000***
SS	3.483 ± 0.832	3.694 ± 0.899	3.619 ± 0.937	NS	3.48 ± 0.83	3.46 ± 1.00	3.70 ± 1.23	NS
RS	2.150(1.7,2.5)	1.800(1.3,2.3)	2.100(1.5,2.5)	NS	3.300(2.0,4.2)	3.300(2.0,4.2)	1.950(1.3,2.7)	0.003***
FS	27.61 ± 2.16	27.68 ± 2.26	26.89 ± 3.80	NS	27.61 ± 2.16	28.57 ± 2.35	28.66 ± 2.71	NS
FD	9.700(8.3,10.8)	9.300(8.8,9.9)	9.350(8.5,10.7)	NS	9.700(8.3,10.8)	10.100(9.1,11.1)	10.000(9.2,10.8)	NS
NA	16.653 ± 7.486	17.552 ± 5.809	16.364 ± 7.049	NS	16.65 ± 7.49	16.34 ± 5.54	17.06 ± 5.99	NS
ASL	4.68 ± 1.06	4.29 ± 1.06	4.32 ± 1.08	NS	4.68 ± 1.06	4.19 ± 1.23	4.49 ± 1.03	NS
PSL	6.867 ± 1.624	7.003 ± 1.325	6.303 ± 1.725	NS	6.87 ± 1.62	7.26 ± 1.20	7.23 ± 2.01	NS
APA	93.864 ± 9.343	92.658 ± 9.101	94.514 ± 8.962	NS	93.86 ± 9.34	92.25 ± 8.79	91.70 ± 11.18	NS

*NS* Not significant

1.*P < 0.05; **P < 0.01; ***P < 0.001; Multiple comparisons were used to analyze the intergroup difference at the level of α = 0.05

### Craniocervical posture in BN, ADDWR and ADDWoR groups

Compared with BN, there were significance differences in all cervical posture measurement items except C1-C2. This suggests that ADD have increased cervical curvature and cervical inclination, indicating that ADD may antevert the upper cervical spine through some unclearly certain mechanisms. However, this change mainly appeared in the upper second cervical vertebra and scarcely appeared in third cervical vertebra possibly because the former is more susceptible (Table 5).

**Table 5:** Comparison of craniocervical posture variables among the BN, ADDWR and ADDWoR groups

Variable	BN	ADDWR	ADDWoR	*P*
Ba-C3ia	5.580(5.3,6.7)	6.420(6.0,6.8)	6.490(6.3,7.2)	0.004**
C0-C1	1.10 ± 0.30	0.75 ± 0.34	0.68 ± 0.23	0.000**
C1-C2	0.660(0.5,0.8)	0.740(0.6,0.9)	0.805(0.6,1.1)	NS
NSL-CP	94.290(87.4,98.5)	98.930(93.7,103.5)	101.275(99.6,108.7)	0.000**
NSL-OPT	96.26 ± 7.73	102.03 ± 8.01	106.48 ± 7.74	0.000**
NL-OPT	86.920(81.0,90.8)	90.800(86.9,96.6)	96.930(92.3,101.4)	0.000**
FH-OPT	87.31 ± 7.61	92.07 ± 7.61	95.13 ± 7.44	0.000**
ML-OPT	115.45 ± 6.46	111.58 ± 7.49	108.82 ± 8.25	0.003**
RL-OPT	177.180(170.2,177.9)	174.170(170.4,178.0)	171.200(165.1,174.9)	0.046*
NSL-C2’	21.66 ± 7.77	26.54 ± 7.44	28.59 ± 9.12	0.004**
MGP-OP	104.15 ± 8.40	99.73 ± 7.34	96.08 ± 7.27	0.000**

*NS* Not significant

1.*P < 0.05; **P < 0.01; ***P < 0.001; Multiple comparisons were used to analyze the intergroup difference at the level of α = 0.05

### Correlation between TMJ and craniocervical posture in ADD groups

The results of Perason correlation analysis between TMJ and craniocervical posture in ADD patients (ADDWR and ADDWoR) showed that MS was significantly correlated with C0-C1, NSL-CP, NSL-OPT, NL-OPT and NSL-C2 (*P* < 0.05), and AS was significantly correlated with NSL-CP, NL-OPT, ML-OPT and NSL-C2 (*P* < 0.05). This suggested that upward and posteromedial displacement of the condyle in the articular fossa is more likely to cause craniocervical posture changed (Table 6).

**Table 6:** Correlations between TMJ and craniocervical posture in ADD groups

Variable	Correlation									
TMJ	Ba-C3ia	C0-C1	NSL-CP	NSL-OPT	NL-OPT	FH-OPT	ML-OPT	RL-OPT	NSL-C2	MGP-OP
AD	NS	0.2^b^	NS	NS	NS	NS	NS	NS	NS	NS
IS	NS	NS	NS	-0.169^a^	-0.148^a^	NS	NS	NS	NS	NS
MS	NS	0.17^a^	-0.226^b^	-0.214^b^	-0.195^b^	-0.165^a^	NS	NS	-0.189^b^	NS
AS	NS	NS	0.05^a^	NS	0.024^a^	NS	-0.196^b^	NS	0.174^a^	NS
RS	NS	NS	NS	NS	NS	NS	NS	NS	NS	NS

*NS* Not significant

^a^Pearson correlation is significant at the 0.05 level

^b^Pearson correlation is significant at the 0.01 level

### Respective correlation between TMJ and craniocervical posture in BN, ADDWR and ADDWoR groups

#### Correlation between TMJ and craniocervical posture in BN group

According to the Pearson correlation analysis, IS was significantly correlated with C0-C1, FH-OPT (*P* < 0.05), AD was significantly correlated with NL-OPT (*P* < 0.05) and RS was significantly correlated with C0-C1 (*P* < 0.05). This indicated that CS posture of healthy people with bilateral normal joint disc position is significantly correlated with three-dimensional movement of the condyle and condylar anteroposterior diameter (Table 7).

**Table 7:** Correlation between TMJ and craniocervical posture in BN group

Variable	Correlation									
TMJ	Ba-C3ia	C0-C1	NSL-CP	NSL-OPT	NL-OPT	FH-OPT	ML-OPT	RL-OPT	NSL-C2	MGP-OP
AD	NS	NS	NS	NS	0.266^a^	NS	NS	NS	NS	NS
IS	NS	-0.259^a^	NS	NS	NS	-0.259^a^	NS	NS	NS	NS
MS	NS	NS	NS	NS	NS	NS	NS	NS	NS	NS
AS	NS	NS	NS	NS	NS	NS	NS	NS	NS	NS
RS	NS	-0.27^a^	NS	NS	NS	NS	NS	NS	NS	NS

*NS* Not significant

^a^Pearson correlation is significant at the 0.05 level

### Correlation between TMJ and craniocervical posture in ADDWR group

According to the Pearson correlation analysis, IS was significantly correlated with NSL-CP, NL-OPT, FH-OPT, and RL-OPT (*P* < 0.05), AS was significantly correlated with C0-C1, NSL-CP, NSL-OPT, NL-OPT (*P* < 0.05) and Ba-C3ia, FH-OPT (*P* < 0.01). Additionally, AD is uncorrelated with CS posture (*P* > 0.05). This implied that CS posture was negatively correlated with IS and AS, positively correlated with RS and not correlated with AD. In other words, CS posture of ADDWR is significantly correlated with condylar three-dimensional movement (mainly sagittal plane) not morphological changes (Table 8).

**Table 8:** Correlation between TMJ and craniocervical posture in ADDWR group

Variable	Correlation									
TMJ	Ba-C3ia	C0-C1	NSL-CP	NSL-OPT	NL-OPT	FH-OPT	ML-OPT	RL-OPT	NSL-C2	MGP-OP
AD	NS	NS	NS	NS	NS	NS	NS	NS	NS	NS
IS	NS	NS	NS	-0.258^a^	-0.272^a^	-0.245^a^	NS	-0.26^a^	NS	NS
MS	NS	NS	NS	NS	NS	NS	NS	NS	NS	NS
AS	-0.381^b^	0.3^a^	-0.251^a^	-0.265^a^	-0.283^a^	-0.39^b^	NS	NS	NS	NS
RS	0.257^a^	0.358^a^	NS	NS	NS	NS	NS	NS	NS	NS

*NS* Not significant

^a^Pearson correlation is significant at the 0.05 level

^b^Pearson correlation is significant at the 0.01 level

### Correlation between TMJ and craniocervical posture in ADDWoR group

According to the Pearson correlation analysis, AD was significantly correlated with C0-C1, ML-OPT and RL-OPT (*P* < 0.05), while MS was significantly correlated with NSL-C2 (*P* < 0.05). CS posture of ADDWoR patients was significantly correlated with condylar morphological changes not condylar three-dimensional movement. The condylar anteroposterior diameter is the primarily responsible for CS posture changes (Table 9).

**Table 9:** Correlation between TMJ and craniocervical posture in ADDWoR group

Variable	Correlation									
TMJ	Ba-C3ia	C0-C1	NSL-CP	NSL-OPT	NL-OPT	FH-OPT	ML-OPT	RL-OPT	NSL-C2	MGP-OP
AD	NS	0.273^a^	NS	NS	NS	NS	-0.285^a^	-0.276^a^	NS	NS
IS	NS	NS	NS	NS	NS	NS	NS	NS	NS	NS
MS	NS	NS	NS	NS	NS	NS	NS	NS	0.235^a^	NS
AS	NS	NS	NS	NS	NS	NS	NS	NS	NS	NS
RS	NS	NS	NS	NS	NS	NS	NS	NS	NS	NS

*NS* Not significant

^a^Pearson correlation is significant at the 0.05 level

^b^Pearson correlation is significant at the 0.01 level

## Discussion

Long-term follow-up surveys have found that the predilection age of TMD is 18–44 years and female patients are about 2.24–5 times higher than male patients [10]. The severity of degenerative changes in TMD increases with age [11], so in order to reduce age-related effects [12], this study restricted patients' age to 18—44 years and female subjects are 3 times more than males.

The current gold standard for diagnosing disc displacement is magnetic resonance imaging (MRI) [13–15], but it is not required for all TMD diagnoses. It is sometimes required to combine imaging examination in order to provide an accurate and comprehensive diagnosis or a definitive diagnosis.Therefore, the manuscript's diagnostic approach is to firstly create a relatively reliably classified and diagnosed TMD patients by RDC/TMD (Research Diagnostic Criteria for TMD) [16]. When the patient's medical history and clinical examination fulfill the RDC/TMD, the various categorization diagnoses is high of specificity, indicating that the diagnosis made is reliable [17]. Relevant literature indicated that its accuracy has excellent consistency to MRI diagnosis of ADDWR or ADDWoR [18, 19]. Secondly, the disc position can be directly confirmed by the the joint space changes for existing literature have demonstrated that the the anterior, superior, and posterior joint space distances measured by CBCT are related to the position of the disc [20–22]. Therefore, this study concluded that the diagnostic approach is dependable of combining TMJ imaging correspond with the latest expert diagnostic consensus of CBCT [23] and clinical symptoms in accordance with RDC/TMD clinical diagnosis meanwhile.

Compared with two-dimensional images, three-dimensional images can more intuitively and simply study the TMJ changes of ADD in detail [24–26], so this study selected the maximum cross-sectional area section of the condyle from the coronal plane, sagittal plane, and horizontal plane for measurement.

In the BN group, the morphology and position of bilateral TMJs were symmetrical, suggesting that there was no significant difference in the bilateral TMJs of people without maxillofacial deformities or joint diseases. And there were no significant differences in bilateral joint fossa width (FS), joint fossa depth (FD), middle joint space (MS) and extra-articular space (ES) in ADDWR and ADDWoR groups. But in general, pathological displacement of the condyles mainly occurred in the ADDWR stage, while organic destruction of the condyles mainly occurred in the ADDWoR stage and bilateral lesions destruction was inconsistently degree. This suggests that bilateral TMJ symmetrical and dynamic changes occur from ADDWR to ADDWoR, as well joint space changes as condylar organic destruction. Normal condylar structures always maintain a convex shape and any condylar structure changes may be attributed to changes in the position of the articular disc [27]. And a number of studies have found a significant correlation between condylar shape, volume, and location with ADD [28, 21]. And as ADD deteriorates from ADDWR to ADDWoR, the size, surface area, and volume of the condyle decrease in all patients [29], which is consistent with the results of this study.

In this study, we found that the anteroposterior diameter of the condyle (AD) gradually decreased during the development from ADDWR to ADDWoR. Kurita et al. [30] showed that condylar width became smaller as ADD progressed, but condylar length did not change significantly, which is consistent with the results of this study. In this study, condylar position and joint space is significant changes [31]. Patients with ADD usually show condylar posterior shift and enlarged anterior joint space (AS) [32]. Condylar posterior shift usually present an enlarged anterior joint space (AS) [33], both of which are important manifestations of ADD [34], which is consistent with this study. In this study, joint space especially AS is significant increscent.

Gateno et al. [35] found that the condylar position of ADD were significantly displaced posteriorly and superiorly, and more posteriorly, which is consistent with the results of this study. Both ADDWR and ADDWoR groups showed significantly anterior joint space (AS) became larger and posterior joint space(RS) became smaller in the bilateral TMJ, which usually means ADD. In addition, intra-articular space (IS) and middle joint space (MS) became smaller. In summary, the condyles tend to be displaced more closer to the articular fossa in ADD patients, which may be one of the pathological mechanisms of TMJ symptoms.

Existing literature reports that cervical spine posture is significantly influenced by the disc and can be altered according to the disc displacement status [36, 37]. The relationship between disc displacement and extended craniocervical posture can be explained in two ways, Firstly, disc displacement leads to significant impairment of vertical and horizontal mandibular growth [38], which in turn may reduce upper airway space. Therefore, extended craniocervical posture may result from protective responses to maintain upper airway space [39]. The second possibility is that extended craniocervical posture may lead to TMJ disc displacement. Previous studies explains it in this way: As the craniocervical posture extend, the mandibular dentition will rotated backward and located more posteriorly in relation to the maxillary dentition. Increased muscular activity that develops as a result will lead to TMJ disc displacement [40, 41]. Therefore, patients with ADD usually show Forward Head Position (FHP) and lower hyoid bone position to compensate for the effects of airway space, caused by mandibular retrusion [42–45].

Based on the above literature’s interpretation, respective correlation analysis of ADD and CS posture of BN, ADDWR and ADDWoR groups were worked. This study's another crucial conclusion reveled that not only condylar pathological movement and morphological changes of ADD patients are significantly related to extended CS posture, but also the relation with CS posture and anterior disc displacement is consistent with the main TMJ pathological characteristic in the current stage of ADD. This could be interpreted as in the ADDWR stage the main pathological characteristic is forced condylar posterior displacement and TMJ space changes and in the ADDWoR stage is morphological change (shape and dimension lesions) [46–48].

## Conclusion

1. In BN group, the morphology and position of bilateral TMJ were overall symmetrical, but not for the ADD groups (the sagittal symmetry is especially more obvious).

2. Compared with BN group, the joint fossa width (FS) and joint fossa depth (FD) of ADDWR and ADDWoR didn’t change significantly, but the condylar anteroposterior diameter (AD) was significantly reduced, the condyle was significantly displaced posteriorly and superiorly, and this change was aggravated with the severity of ADD stage, which may be one of the causes of joint symptoms of TMD.

3. Disc displacement was significantly correlated with craniocervical posture, and the degree increased with the progression of ADD.

4. Bilateral TMJ of ADD patients mainly occurs condylar shape and position changes and condylar structural damages. The progression of ADD will further aggravate the extension of craniocervical posture.

5. Condylar pathological movement and morphological changes are significantly related to extended CS posture and the relation is consistent with the main pathological characteristic of the current stage of ADD.

### Deficiencies and prospects

This study has the following four shortcomings. First, as the disc displacement progresses from ADDWR to ADDWoR, the condylar position varies greatly, and the specific changes need to be further studied by increasing the sample size. Second, in order to provide more reliable clinical bases, long-term stability of TMJ changes requires perennial clinical follow-up. Thirdly, the causal relationship between disc displacement and craniocervical postural anteversion is not clear because the results were derived from cross-sectional data, therefore a cohort study should be used to investigate the differences in craniocervical posture between the population with and without anterior disc displacement to further investigate the causal relationship. Fourthly, The measurement results of cervical craniocervical posture are based on lateral cephalogram under static posture, therefore it’s not possible to conduct in-depth research on the relationship between condylar movement and mandibular load with craniocervical posture, as well as functional state detection related to mandibular dynamics.

### Supplementary Information

**Additional file 1.**

## Data Availability

All data and materials in this study are available by contacting correspondence.

## References

[1] Durham J, Newton-John TR, Zakrzewska JM (2015). Temporomandibular disorders. BMJ.

[2] Kundu H, Basavaraj P, Kote S (2013). Assessment of TMJ disorders usingultrasonography as a diagnostic tool: a re-view. J Clin Diagn Res.

[3] Slade GD, Fillingim RB, Sanders AE (2013). Summary of findings from the OPPERA prospective cohort study of incidence of first-onset temporomandibular disorder: implications and future directions. J Pain.

[4] Wadhwa S, Kapila S (2008). TMJ disorders: future innovations in diagnostics and therapeutics. J Dent Educ.

[5] Poluha RL, Canales GT, Costa YM (2019). Temporomandibular joint disc displacement with reduction: a review of mechanisms and clinical presentation. J Appl Oral Sci.

[6] Sonnesen L, Pedersen CE, Kjaer I (2007). Cervical column morphology related to head posture, cranial base angle, and condylar malformation. Eur J Orthod.

[7] Olivo SA, Bravo J, Magee DJ, Thie NM, Major PW, Flores-Mir C (2006). The association between head and cervical posture and temporomandibular disorders: a systematic review. J Orofac Pain.

[8] Ciancaglini R, Colombo-Bolla G, Gherlone EF (2003). Orientation of craniofacial planes and temporomandibular disorder in young adults with normal occlusion. J Oral Rehabil.

[9] Bueno CH, Pereira DD, Pattussi MP (2018). Gender differences in temporomandibular disorders in adult population studies: A systematic review and meta-analysis. J Oral Rehabil.

[10] Alexiou K, Stamatakis H, Tsiklakis K (2009). Evaluation of the severity of temporomandibular joint osteoarthritic changes related to age using cone beam computed tomography. Dentomaxillofac Radiol.

[11] Basaran M, Bozdemir E, Evrimler S (2021). Evaluation of morphometric and volumetric measurements of temporomandibular joint structures on patients with disc displacement. J Anat Soc India.

[12] Yang Z, Wang M, Ma Y (2017). Magnetic resonance imaging (MRI) evaluation for anterior disc displacement of the temporomandibular joint. Med Sci Monit.

[13] Sinha VP, Pradhan H, Gupta H (2012). Efficacy of plain radiographs, CT scan, MRI and ultrasonography in temporomandibularjoint disorders. Natl J Maxillofac Surg.

[14] Weinberg LA (1970). An evaluation of duplicability of temporomandibular joint radiographs. J Prosthet Dent.

[15] Schiffman E, Ohrbach R, Truelove E, etc. Diagnostic Criteria for Temporomandibular Disorders (DC/TMD) for Clinical and Research Applications: recommendations of the International RDC/TMD Consortium Network* and Orofacial Pain Special Interest Group†. J Oral Facial Pain Headache. 2014 Winter;28(1):6–27. doi: 10.11607/jop.1151. PMID: 24482784; PMCID: PMC4478082. 10.11607/jop.1151 PMC4478082 24482784

[16] Fu KY (2017). Interpretation of newly published (2014) diagnostic criteria for temporomandibular disorders (DC/TMD). Zhonghua Kou Qiang Yi Xue Za Zhi..

[17] Manfredini D, Guarda-Nardini L (2008). Agreement between Research Diagnostic Criteria for Temporomandibular Disorders and magnetic resonance diagnoses of temporomandibular disc displacement in a patient population. Int J Oral Maxillofac Surg.

[18] Park JW, Song HH, Roh HS (2012). Correlation between clinical diagnosis based on RDC/TMD and MRI findings of TMJ internal derangement. Int J Oral Maxillofac Surg.

[19] Schnabl D, Rottler AK, Schupp W (2018). CBCT and MRT imaging in patients clinically diagnosed with temporomandibular joint arthralgia. Heliyon.

[20] Dumas ALMRM, Willis HB, Homayoun NM (2017). Assessment of condyle position, fossa morphology, and disk displacement in symptomatic patients. Oral Surg Oral Med Oral Pathol Oral Radiol.

[21] Mohamed HN, Ashmawy MS, Ekladious MEY (2023). Analysis of the relationship between condylar changes and anterior disc displacement with reduction: a preliminary study. Oral Radiol.

[22] Fu KY, Hu M, Yu Q (2020). Experts consensus on cone-beam CT examination specification and diagnostic criteria of temporomandibular disorders. Zhonghua Kou Qiang Yi Xue Za Zhi..

[23] Dupuy-Bonafé I, Otal P, Montal S (2014). Biometry of the temporomandibular joint using computerized tomography. Surg Radiol Anat.

[24] Zhang YL, Song JL, Xu XC (2016). Morphologic Analysis of the Temporomandibular Joint Between Patients With Facial Asymmetry and Asymptomatic Subjects by 2D and 3D Evaluation: A Preliminary Study. Medicine (Baltimore).

[25] Praveen Bn SH. Morphological and radiological variations of mandibular condyles in health and diseases a systematic review. Dentistry. 2013. 10.4172/2161-1122.1000154.

[26] McNeill C, Mohl ND, Rugh JD, et al. Temporomandibular disorders: diagnosis, management, education, and research. J Am Dent Assoc. 1990 Mar;120(3):253, 255, 257 passim. doi: 10.14219/jada.archive.1990.0049. PMID: 2179355. 10.14219/jada.archive.1990.0049 2179355

[27] Ma RH, Feng JL, Bornstein MM, Li G (2023). Relationship between development of the condylar cortex and the changes in condyle morphology: a cone-beam computed tomography (CBCT) observational study. Quant Imaging Med Surg.

[28] Rabelo KA, Sousa Melo SL, Torres MGG (2017). Assessment of condyle position, fossa morphology, and disk displacement in symptomatic patients. Oral Surg Oral Med Oral Pathol Oral Radiol.

[29] Katzberg RW, Keith DA, Ten Eick WR (1983). Internal derangements of the temporomandibular joint: an assessment of condylar position in centric occlusion. J Prosthet Dent.

[30] Kurita H, Ohtsuka A, Kobayashi H (2002). Alteration of the horizontal mandibular condyle size associated with temporomandibular joint internal derangement in adult females[J]. Dentomaxillofac Radiol.

[31] Seo BY, An JS, Chang MS, Huh KH, Ahn SJ (2020). Changes in condylar dimensions in temporomandibular joints with disk displacement. Oral Surg Oral Med Oral Pathol Oral Radiol.

[32] Dumas ALMRM, Willis HB, Homayoun NM (1984). A tomographic study of the condyle/fossa relationship in patients with TMJ dysfunction. J Craniomand Pract.

[33] Weinberg LA (1979). Role of condylar position in TMJ dysfunction-painsyndrome. J Prosthet Dent.

[34] Bonilla-Aragon H, Tallents RH, Katzberg RW (1999). Condyle position as a predictor of temporomandibular joint internal de-rangement. J Prosthet Dent.

[35] Gateno J, Anderson PB, Xia JJ (2004). A comparative assessment of mandibular condylar position in patients with anterior disc displacement of the temporomandibular joint. J Oral Maxillofac Surg.

[36] de Farias Neto JP, de Santana JM, de Santana-Filho VJ, Quintans-Junior LJ, de Lima Ferreira AP, Bonjardim LR (2010). Radiographic measurement of the cervical spine in patients with temporomandibular dysfunction. Arch Oral Biol.

[37] Huggare JA, Raustia AM (1992). Head posture and cervicovertebral and craniofacial morphology in patients with craniomandibular dysfunction. Cranio.

[38] Solow B, Sandham A (2002). Cranio-cervical posture: a factor in the development and function of the dentofacial structures. Eur J Orthod.

[39] Nicolakis P, Nicolakis M, Piehslinger E, et al. Relationship between craniomandibular disorders and poor posture. Cranio 2000;18:106–12.; [34]Ansar J, Maheshwari S, Verma SK, et al: Soft tissue airway dimensions and craniocervical posture in subjects with different growth patterns. Angle Orthod 85:604:2015 10.2319/042314-299.1 PMC8611761 25245417

[40] Ceylan I, Oktay H (1995). A study on the pharyngeal size in different skeletal patterns. Am J Orthod Dentofacial Orthop.

[41] Grauer D, Cevidanes LS, Styner MA (2009). Pharyngeal airway volume and shape from cone-beam computed tomography: Rela-tionship to facial morphology. Am J Orthod DentofacialOrthop.

[42] Ahn SJ, Kim TW, Nahm DS (2004). Cephalometric keys to internal derangement of temporomandibular joint in women with Class II malocclusions. Am J Orthod.

[43] Kang JH, Sung J, Song YM, Kim YH (2018). Heritability of the airway structure and head posture using twin study. J Oral Rehabil.

[44] An JS, Jeon DM, Jung WS (2015). Influence of temporomandibular joint disc displacement on craniocervical posture and hyoid bone position. Am J Orthod Dentofacial Orthop.

[45] Adamidis IP, Spyropoulos MN (1992). Hyoid bone position and orientation in Class I and Class III malocclusions. Am J Orthod Dentofacial Orthop.

[46] Seo BY, An JS, Chang MS (2020). Changes in condylar dimensions in temporomandibular joints with disk displacement. Oral Surg Oral Med Oral Pathol Oral Radiol.

[47] Ahn SJ, Chang MS, Choi JH (2018). Relationships between temporomandibular joint disc displacements and condylar volume. Oral Surg Oral Med Oral Pathol Oral Radiol.

[48] Rao VM, Babaria A, Manoharan A (1990). Altered condylar morphology associated with disc displacement in TMJ dysfunction: observations by MRI. Magn Reson Imaging.

