# Supplementary material for: Correlation between temporomandibular joints and craniocervical posture in patients with bilateral anterial disc displacement
Source: BMC Oral Health. 2024 Feb 1;24:159. doi: 10.1186/s12903-024-03892-9 (PMC10832266; doi:10.1186/s12903-024-03892-9)
Supplement: Supplementary file 1 — Additional file 1. [file 12903_2024_3892_MOESM1_ESM.zip › Supplementary material/1. Supplemental data.docx]

**Supplemental Data**

**Supplementary table S1.** The inclusion and exclusion criteria of subjects

| **Inclusion criteria:** |
| --- |
| 1) Subjects with TMD corresponding symptoms (pain, clicking, functional limitation, etc.);  2) Diagnosed with bilateral anterior disc displacement by joint specialists;  3) The full dentition is basically neat, without bad masticatory habits or night bruxism symptoms;  4) The patient was healthy, permanent dentition, the peak growth and development ended, and the body mass index was normal. |
| **Exclusion criteria:** |
| 1) Patients with autoimmune or underlying diseases such as rheumatoid arthritis and hypertensive diabetes;  2) patients with more than two missing teeth, elongated of jaw teeth, or no tooth loss but elongated wisdom teeth;  3) History of craniomaxillofacial deformity, cleft lip and palate and facial deformity surgery;  4) history of temporomandibular joint trauma or surgery; |

**Supplementary table S2.** Sample size power (by PASS 15.0.5 software calculation)

The results showed that when the sample size n = 90 (significance level *p* = 0.05), the test efficacy of one - way ANOVA was 81.65 %, indicating that the sample size can meet the statistical analysis requirements of this experiment.


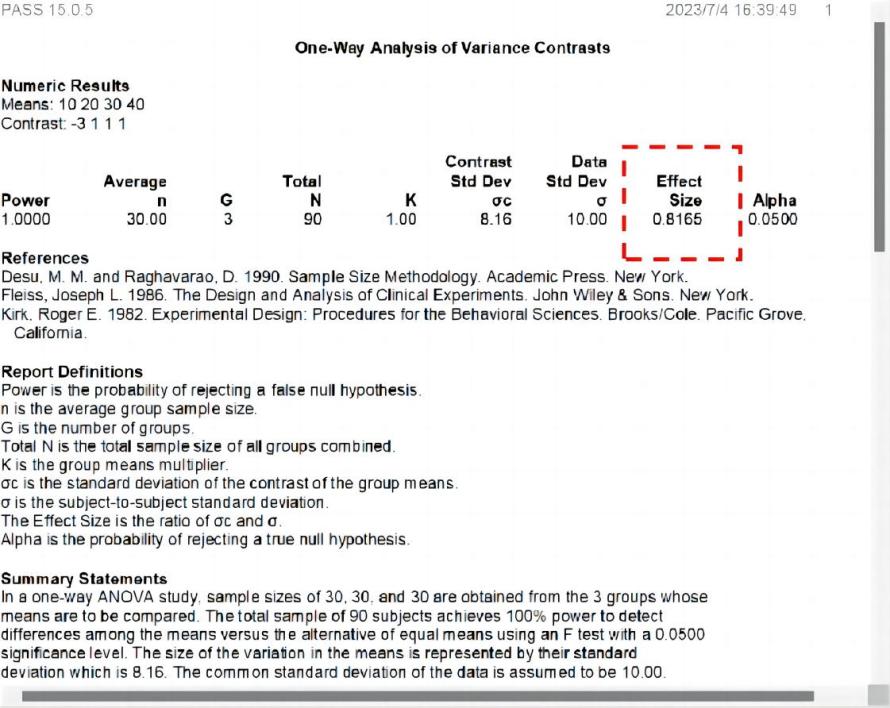


**Supplementary table S3.** The Abbreviation and its definition

| **Abbreviation** | **Definition** |
| --- | --- |
| TMJ | Temporomandibular joint |
| TMD | Temporomandibular disorders |
| ADDWR | Anterior disc displacement with reduction |
| BN | Bilateral Disc Normal Position |
| ADDWoR | Anterior Disc Displacement Without Reduction |
| CS | Cervical Spine |
| CBCT | Cone-beam computed tomography |
| FHP | Forward head posture |
| MRI | Magnetic Resonance Imaging |
| CV | Cervical Vertebrae |

**Supplementary table S4.** The RDC/TMD Diagnostic Criteria for ADDWR and ADDWoR

| Classification | Clinical Diagnostic Criteria |
| --- | --- |
| ADDWR | Medical history of joint sounds, or patient reporting joint sounds during examination. A popping sound is heard during open and close mouth movements, or between open and close mouth movements and lateral or forward extension movements, according to clinical examination. |
| ADDWR **with strangulation** | The clinical examination was performed with the mouth open and closed, or with opening and closing and side or front stretch motions; if the joint hangs during the test, the technique can be aided by opening. |
| ADDWR **with limited opening** | A history or claim of an underlying lock or blockage (even short-term locking or blocking) now limits the opening of the seat lock and has an impact on the diet.Clinical examination passive mouth openness 40 mm |
| ADDWR **without limited opening** | A history of restricted section lock opening and influenced eating, as well as an allegation of an underlying lock or blockage (including short-term locking or blocking).Clinical examination passive mouth opening 40 mm |
